# DT-13 synergistically enhanced vinorelbine-mediated mitotic arrest through inhibition of FOXM1-BICD2 axis in non-small-cell lung cancer cells

**DOI:** 10.1038/cddis.2017.218

**Published:** 2017-05-25

**Authors:** Hongyang Li, Li Sun, Hang Li, Xiaodan Lv, Herve Semukunzi, Ruiming Li, Jun Yu, Shengtao Yuan, Sensen Lin

**Affiliations:** 1 Jiangsu Center for Pharmacodynamics Research and Evaluation, China Pharmaceutical University, Nanjing 210009, China; 2 Jiangsu Key Laboratory of Drug Screening, China Pharmaceutical University, Nanjing 210009, China; 3 Tasly Research Institute, Tianjin Tasly Holding Group Co. Ltd, Tianjin 300410, China; 4 Jiangsu Cancer Hospital, Nanjing 210009, China

## Abstract

Non-small-cell lung cancer (NSCLC) is the most commonly diagnosed malignant disease with the leading cause of cancer-related death. Combination treatment remains the major strategy in the clinical therapy of NSCLC. Vinorelbine (NVB), a semi-synthetic vinca alkaloid, is used for advanced and metastatic NSCLC by destabilizing microtubule formation to induce mitotic arrest and cell death. However, the side effect of NVB heavily affected its effectiveness in clinical therapy. Hence, it is of great significance to develop new agents to synergize with NVB and decrease the adverse effect. In our study, we found that the saponin monomer 13 of the dwarf lilyturf tuber, DT-13, exhibiting anti-angiogenesis and anti-metastasis effect, synergized with NVB to inhibit cell proliferation in NSCLC cells. The synergistic interaction of DT-13 and NVB was confirmed by combination Index values. Also, DT-13 and NVB act in concert to inhibit the long-term colony formation. Furthermore, DT-13/NVB co-treatment cooperated to induce mitotic arrest and subsequent apoptosis. Mechanistically, we found that nuclear expression of transcription factors forkhead box M1 (FOXM1) and levels of motor adaptor bicaudal D2 (BICD2) were dramatically reduced by combination treatment. Importantly, oncogene FOXM1 was identified as the crucial regulator of BICD2, which played critical roles in NVB-induced mitotic spindle defects. Moreover, overexpression of FOXM1 and BICD2 significantly reversed mitotic arrest induced by DT-13/NVB co-treatment, and siRNAs against both genes greatly increased the combinational effects. In addition, *in vivo* study revealed that DT-13 combined with NVB significantly suppressed tumor growth in nude mice xenograft model, and downregulated the expression of FOXM1 and BICD2 in tumor tissues, which was consistent with *in vitro* study. In conclusion, DT-13 might provide a novel strategy for the chemosensitization of NVB in NSCLC therapy.

Non-small-cell lung cancer (NSCLC) is most commonly diagnosed and malignant type of lung cancer, which remains the leading cause of tumor-related deaths.^1^ Nowadays, chemotherapy and molecular-targeted drugs are the main therapy option for NSCLC therapy except radiotherapy and surgical resection. Moreover, different epidermal growth factor receptor (EGFR) status in NSCLC displayed various sensitivity of chemotherapy and EGFR inhibitors. Recent studies have demonstrated that chemotherapy was more effective than EGFR inhibitors, gefitinib or erlotinib, to prolong the progression-free survival and overall survival of patients with NSCLC who exhibited wild-type EGFR.^2, 3, 4^ Thus, improving effectiveness of chemotherapy is also of great significance for the particular NSCLC patients.

To ensure genomic stability in cell cycle progression without uncompletely replicated and damaged DNA, eukaryote cells mainly depended on a tightly controlled surveillance program such as G1/S, G2/M and spindle assembly checkpoint (SAC).^5, 6, 7^ Dysregulation in cell cycle transition is a property of cancer development, and disruption of the progression can trigger cell cycle arrest and subsequent cell death, which contributes to cancer suppression.^8^ Microtubule-targeting agents (MTAs), such as taxanes and vinca alkaloids, have gained great success in clinical therapy by activating SAC to induce mitotic arrest. However, clinical toxicity and chemotherapeutic resistance seriously hampered the application and development of these cytotoxic drugs. To overcome these adverse effects, favorable combination strategy is urgently needed to be developed.

Cell cycle progression is partly regulated by multiple transcription factors. Forkhead box M1 (FOXM1), a member of Forkhead family, is an oncogenic transcription factor, and highly expressed in various cancers.^9^ A number of studies have shown that FOXM1 played important roles in cell proliferation, angiogenesis, metastasis, cellular senescence and drug resistance.^9, 10, 11^ In mitosis progression, FOXM1 controlled mitotic entry by regulating Cdc25B, cyclin B, PLK-1 and Nek-2, SAC activation by centromere protein A, B, F (CENP-A, B and F), KIF20A, PLK-1, Aurora A and B, cytokinesis and mitotic exit by Aurora-B, Plk-1 and survivin.^11, 12^ Furthermore, FOXM1 expression was also involved in the drug sensitivity and resistance of paclitaxel.^11, 13^ Hence, targeting FOXM1 may be a feasible strategy to improve the effectiveness of MTAs.

Vinorelbine (NVB), as a semi-synthetic vinca alkaloid, is used for the treatment of advanced and metastatic NSCLC by destabilizing microtubule formation and activating SAC to induce mitotic arrest and cell death.^14^ Although it is widely used in clinical application, myelosuppression, neurotoxicities and drug resistance became major obstacle for its clinical application.^15, 16, 17^ DT-13, a saponin monomer 13 of the dwarf lilyturf tuber, was derived from Liriopes Radix.^18^ Our previous research showed that DT-13 exhibited pro-autophagy,^19^ anti-thrombus and anti-inflammation activity.^20, 21^ Furthermore, DT-13 inhibited the cancer cell metastasis,^22^ cancer angiogenesis^23^ and synergistically enhanced topotecan-induced apoptosis.^24^

In our present study, we found that DT-13 combined with NVB exhibited potent synergistic effects to inhibit the proliferation of NSCLC cells according to a set of screening, and further demonstrated that FOXM1 levels were involved in the synergistic effect *in vitro* and *in vivo*.

## Results

### DT-13 synergistically increased the cytotoxicity of NVB in NSCLC cells

**Figure 1:**
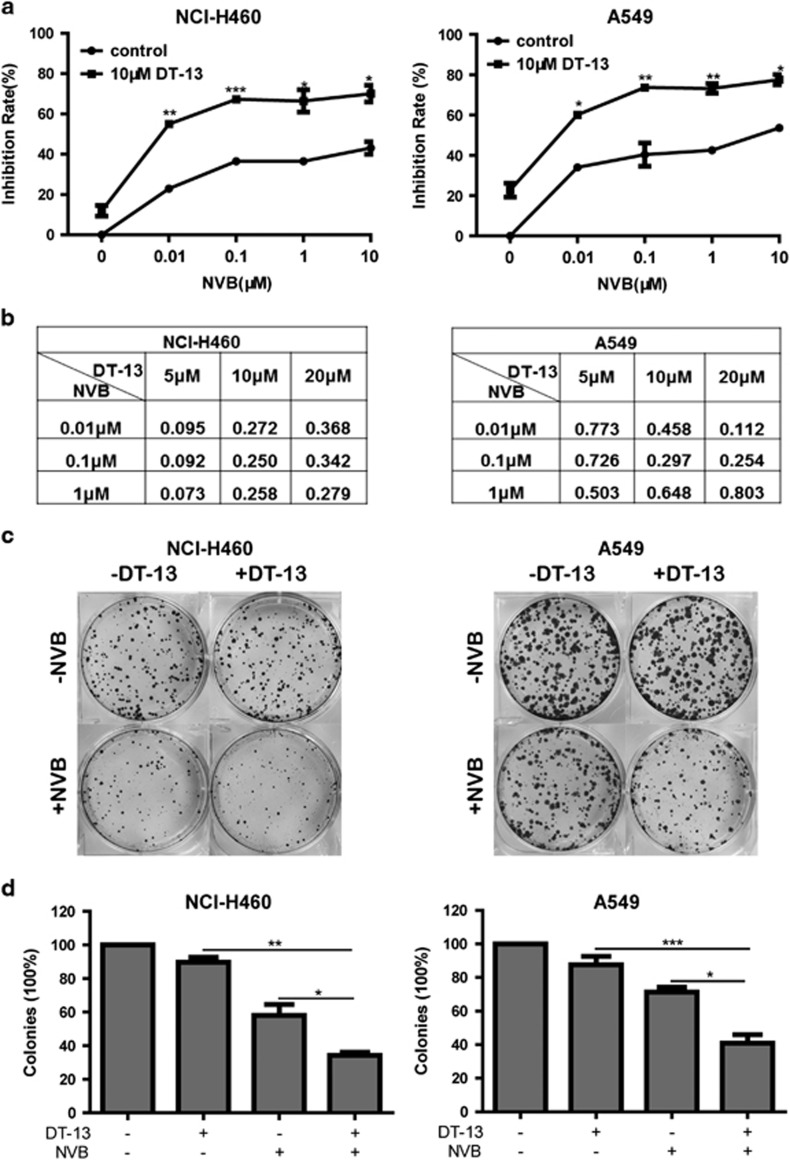
DT-13 and NVB synergized to inhibit cell proliferation in NSCLC cells. (**a**) NCI-H460 and A549 cells were treated with 10 *μ*M DT-13 and indicated concentrations of NVB for 48 h. MTT assays were performed to analyze the cell viability of NSCLC cells. (**b**) CI values were calculated by CalcuSyn software, and drug interactions were indicated as synergism (CI<0.9), additivity (0.9<CI<1.1) or antagonism (CI>1.1). (**c**) NCI-H460 and A549 cells were exposed to 10 *μ*M DT-13 and/or 0.01 *μ*M NVB for 12 h, and incubated for another 8 days. The colonies were stained with crystal violet. (**d**) The number of colonies was counted macroscopically, and expressed as the percentage of untreated control. The data were expressed as mean±S.D. in triplicate using Student’s *t*-test (two-tailed). *P<0.05, **P<0.01 and ***P<0.001

To investigate whether DT-13 can be a candidate exploited to increase the drug sensitivity in NSCLC cells, we explored the combination treatment of DT-13 and NVB, which was used in standard chemotherapy regimen of NSCLC.^25^ As shown in Figure 1a, MTT assay showed that 10 *μ*M DT-13 significantly increased the cytotoxicity of NVB in NCI-H460 and A549 cells. Drug interaction of DT-13 and NVB was calculated by combination Index (CI) values (Figure 1b), which demonstrated that DT-13/NVB co-treatment exhibited potent synergistic effect in NSCLC cells. Furthermore, compared with DT-13 and NVB treatment alone, combination treatment dramatically inhibited the colony formation in both NCI-H460 and A549 cells (Figures 1c and d).

To explore whether the synergistic interaction of DT-13 and NVB was of broader relevance in other NSCLC cells, we used NCI-H1975 and HCC827 cells to evaluate the combinational effects. As shown in Supplementary Figures 1A and B, DT-13/NVB co-treatment also exhibited synergistic effects to inhibit the proliferation of both NCI-H1975 and HCC827 cells. Moreover, taxol, a classical anti-mitosis drugs, was also evaluated in the combination treatment with DT-13. We found that DT-13 synergistically enhanced the cytotoxicity of taxol in NCI-H460, NCI-H1975 and HCC827 cells except for A549 cells (Supplementary Figures 1C–F). Overall, the synergistic interaction of DT-13 and NVB or taxol was summarized in Supplementary Table S1, and the date demonstrated that DT-13 could synergistically enhance NVB or taxol sensitivity in NSCLC cells.

### DT-13 and NVB cooperated to trigger caspase-dependent apoptosis in NSCLC cells

**Figure 2:**
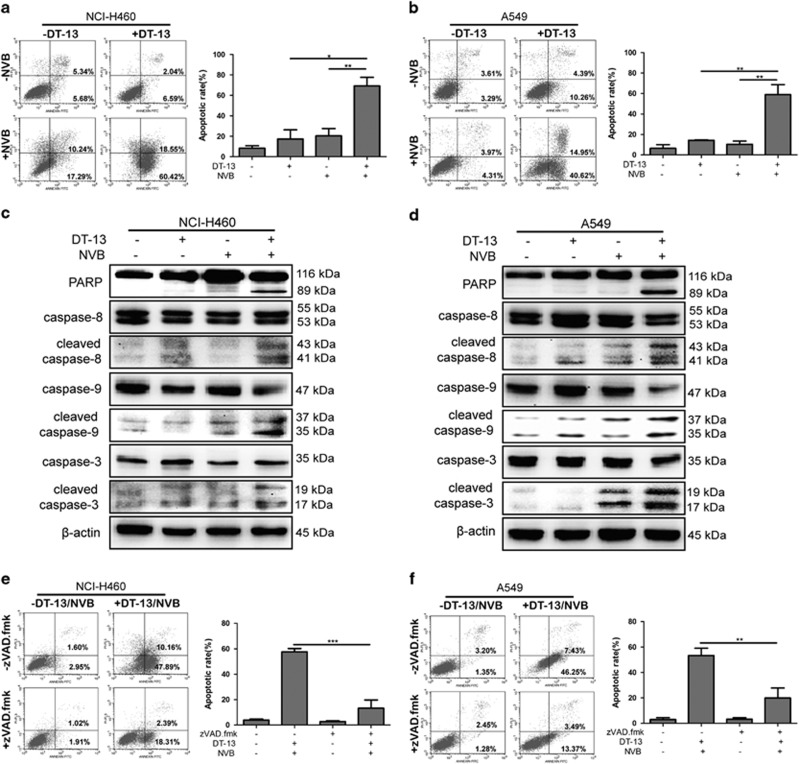
Combination treatment of DT-13 and NVB-induced caspase-dependent apoptosis in NSCLC cells. NCI-H460 and A549 cells were treated with 10 *μ*M DT-13 and 0.01 *μ*M NVB for 48 h. Annexin V/PI analysis for NCI-H460 (**a**) and A549 (**b**) cells were performed by flow cytometry to detect the percentage of apoptotic cells, and the frequency of apoptotic cells (including early and late apoptotic cells) was shown in the histograms. The activation of apoptosis-related proteins for NCI-H460 (**c**) and A549 (**d**) cells at 48 h was observed by the detection of active cleavage fragments of PARP, caspase-8, caspase-9 and caspase-3, *β*-actin was served as the loading control. NCI-H460 (**e**) and A549 (**f**) cells were pretreated with 10 *μ*M zVAD.fmk for 2 h, and then cells were exposed with 10 *μ*M DT-13 and 0.01 *μ*M NVB for another 48 h. Annexin V/PI analysis was performed to detect the percentage of apoptotic cells, and the frequency of apoptotic cells (including early and late apoptotic cells) was shown in the histograms. The data were expressed as mean±S.D. in triplicate using Student’s *t*-test (two-tailed). *P<0.05, **P<0.01 and ***P<0.001

The current study showed that DT-13/NVB co-treatment exhibited synergistic effects in NCI-H460 and A549 cells, both of which displayed wild-type EGFR status. Hence, it is of great significance to investigate the synergistic mechanisms of DT-13 and NVB. NVB has been reported to cause apoptosis by releasing cytochrome *c* and activating caspase-related proteins in NSCLC cells.^26^ To demonstrate whether apoptosis was involved in the synergistic effect, we performed Annexin V/PI staining after DT-13 and NVB co-treatment, and results showed that the combination treatment significantly induced apoptosis in NCI-H460 and A549 cells for 48 h, compared with DT-13 or NVB treatment alone (Figures 2a and b). At a mechanistic level, PARP cleavage and caspases activation were known as important effectors of apoptosis induction.^27^ Western blotting analysis showed that DT-13 and NVB cooperated to induce the cleavage of PARP and the activation of caspase-8, caspase-9 and caspase-3 (Figures 2c and d). To investigate the requirement of caspase activity for apoptosis induction, we applied the broad range caspase inhibitor zVAD.fmk, and found that pretreatment of zVAD.fmk greatly reduced DT-13/NVB-induced apoptosis in both NCI-H460 and A549 cells (Figures 2e and f). Moreover, combination treatment for 48 h triggered significant changes of cell number and morphology, compared with DT-13 or NVB treatment alone (Supplementary Figure 2A). Above all, these data determined that DT-13/NVB co-treatment induced caspase-dependent apoptosis, which was the result of synergistic drug interactions.

### DT-13 dramatically potentiated NVB-caused mitotic arrest in NSCLC cells

**Figure 3:**
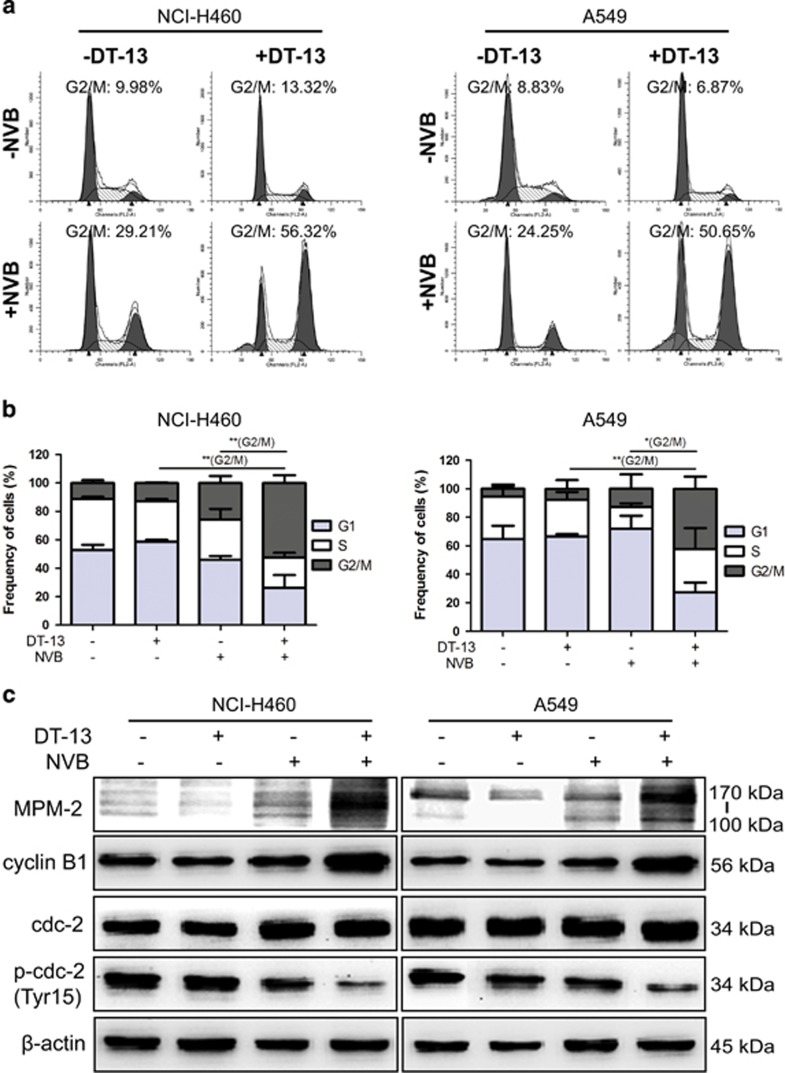
DT-13 promoted the mitotic arrest induced by NVB in NSCLC cells. NCI-H460 and A549 cells were treated with 10 *μ*M DT-13 and 0.01 *μ*M NVB for 12 h. (**a**) Frequency of cells per phase in cell cycle was detected by flow cytometry using PI staining. (**b**) Percentages of cells in each phase were shown in the histograms, and the data were expressed as mean±S.D. in triplicate using Student’s *t*-test (two-tailed) to evaluate the statistical discrepancy of G2/M arrest. *P<0.05, **P<0.01. (**c**) Expression of mitosis-related proteins was analyzed by detection of the expression of MPM2, cyclin B1, cdc2 and phosphorylation of cdc2 (Tyr15), *β*-actin was served as the loading control

As reported, NVB bound to tubulin and induced the depolymerization of microtubule, and SAC was then activated and cells were arrested in mitotic phase.^28^ Here, we found that mitotic arrest was not greatly induced by DT-13/NVB co-treatment in both NCI-H460 and A549 cells at 48 h, but the percentage of sub G1 cells was greatly increased (Supplementary Figure 3), which was consistent with the results of apoptosis induction in Figure 2. In addition, prolonged mitotic arrest could trigger intrinsic apoptotic pathway.^29^ Therefore, we investigated whether DT-13/NVB co-treatment caused mitotic arrest after short-term treatment. As expected, we found that cells treated with the combination of DT-13 and NVB turned rounder (Supplementary Figure 2B), and were greatly arrested in G2/M phase at 12 h (Figure 3a). The percentage of G2/M phase cells in combination treatment was dramatically higher than DT-13 or NVB treatment alone (Figure 3b). To determine that combination treatment induced cell cycle arrest in G2 phase or in mitotic phase, we used MPM2 as the specific mitotic marker.^30^ Western blotting analysis revealed that MPM2 expression was powerfully induced by DT-13 combined with NVB (Figure 3c). Furthermore, the transition from G2 phase to mitosis required the activation of cyclin B1/CDK1 checkpoint complex.^31, 32^ Here, we found that cyclin B1 was accumulated and CDK1/cdc2 was activated by combination treatment (Figure 3c). Collectively, the results demonstrated that DT-13 synergistically potentiated NVB-induced mitotic arrest in NSCLC cells.

### Mitotic arrest induced by combination treatment was necessary for apoptosis induction

**Figure 4:**
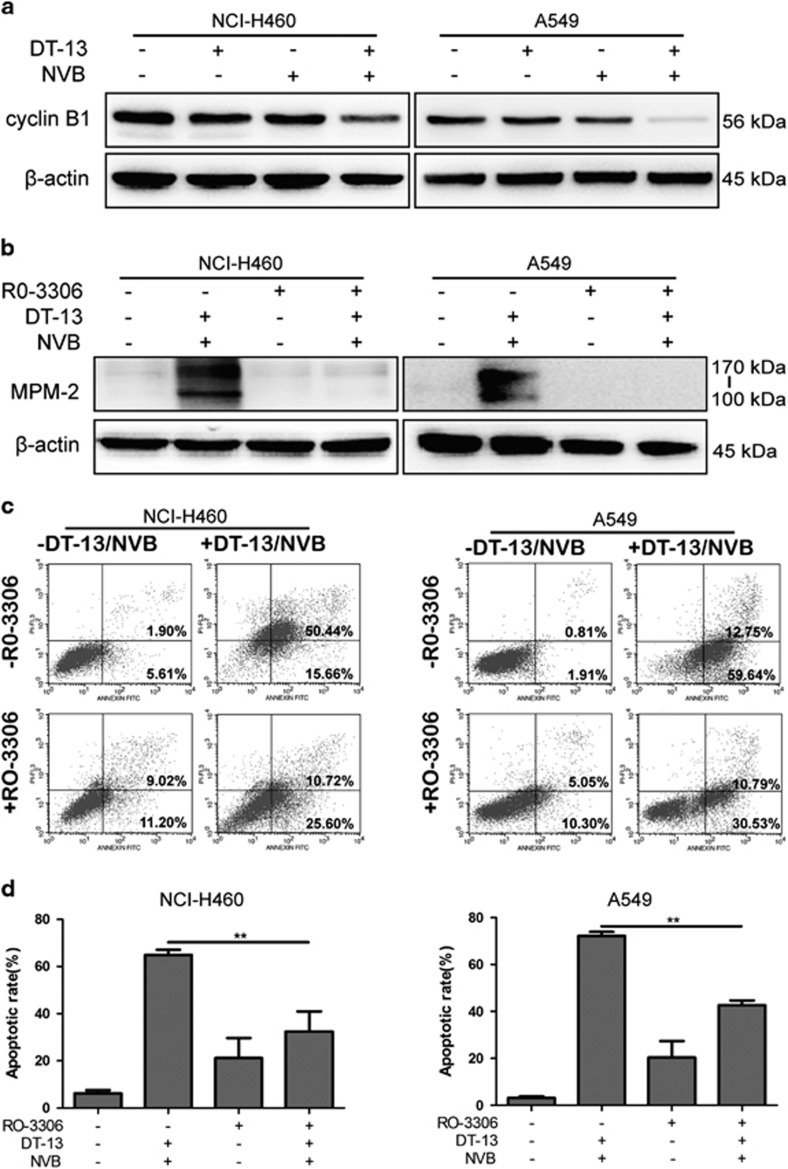
Mitotic arrest induced by DT-13 and NVB was required for apoptosis induction. (**a**) NCI-H460 and A549 cells were treated with 10 *μ*M DT-13 and 0.01 *μ*M NVB for 48 h, and the expression of cyclin B1 was determined by western blotting analysis. (**b**) NCI-H460 and A549 cells were pretreated with 5 *μ*M CDK1 inhibitor RO-3306 for 2 h, and cells were then exposed to 10 *μ*M DT-13 and 0.01 *μ*M NVB for another 12 h. Expression of mitotic marker MPM2 was observed by western blotting, and *β*-actin was served as the loading control. (**c**) Cells were treated with 10 *μ*M DT-13 and 0.01 *μ*M NVB for 48 h after pretreatment of 5 *μ*M RO-3306 for 2 h, and apoptotic cells were determined by Annexin V/PI staining. (**d**) The percentage of apoptotic cells (including early and late apoptotic cells) was shown in the histograms, and the data were expressed as mean±S.D. in triplicate using Student’s *t*-test (two-tailed). **P<0.01

As reported, when levels of cyclin B1 expression decreased below the threshold that required for the activation of CDK, cell death pathway would be activated.^29, 33^ Here, we found that cyclin B1 expression was dramatically increased at short-term treatment (Figure 3c) and decreased at long-term treatment (Figure 4a) in both NCI-H460 and A549 cells. To further demonstrate whether DT-13/NVB-induced mitotic arrest was required for apoptosis induction, CDK1 inhibitor RO-3306 was used to inhibit cell cycle transition from G2 phase to mitosis (Figure 4b). Interestingly, DT-13/NVB-induced apoptosis at 48 h was greatly reduced by pretreatment of RO-3306 (Figures 4c and d). This set of experiments demonstrated that DT-13/NVB-induced mitotic arrest was required for the activation of apoptosis pathway.

### Combination of DT-13 and NVB inhibited FOXM1 and BICD2 expression at mRNA and protein levels

**Figure 5:**
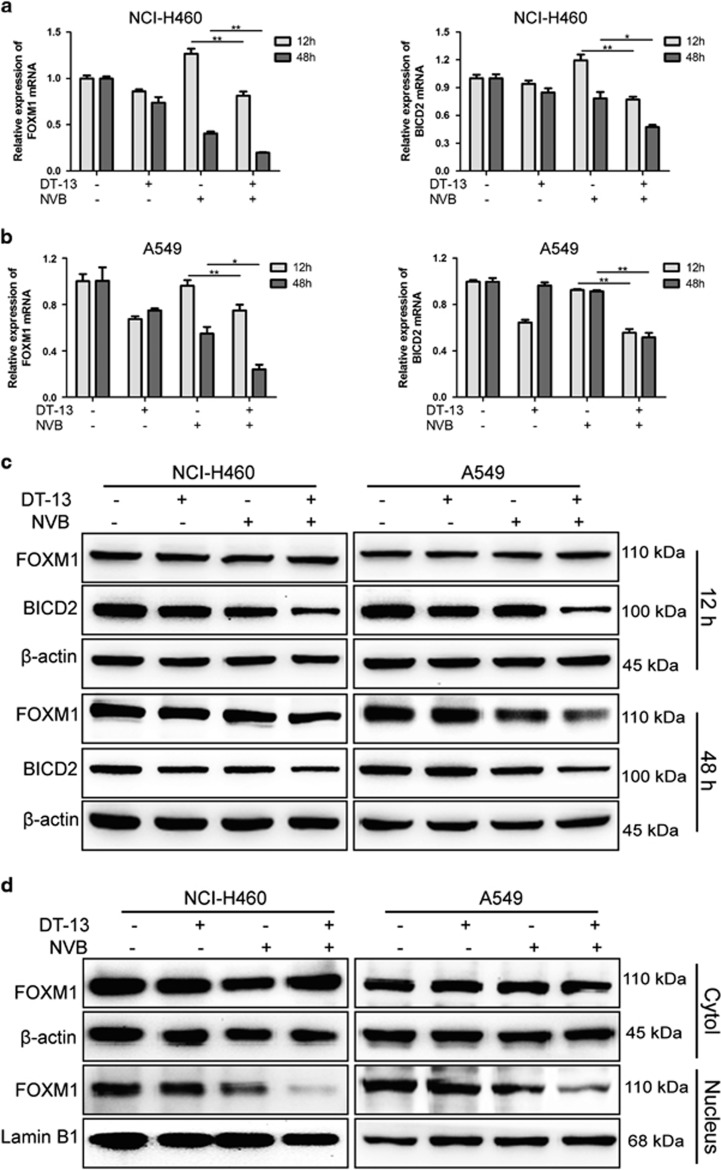
DT-13/NVB co-treatment inhibited the expression of FOXM1 and BICD2 at mRNA and protein levels. NCI-H460 and A549 cells were treated with 10 *μ*M DT-13 and 0.01 *μ*M NVB for 12 and 48 h, respectively. The mRNA levels of FOXM1 and BICD2 in NCI-H460 (**a**) and A549 (**b**) cells were detected by RT-qPCR analysis, and the data were expressed as mean±S.D. in triplicate using Student’s *t*-test (two-tailed). *P<0.05, **P<0.01. (**c**) Protein levels of FOXM1 and BICD2 in NCI-H460 and A549 cells were determined by western blotting analysis, and *β*-actin was served as the loading control. (**d**) Nuclear and cytoplasmic expression of FOXM1 at 12 h was detected by western blotting. Lamin B1 and *β*-actin were served as the loading control in nucleus and cytoplasm, respectively. RT-qPCR, real-time quantitative PCR

As reported, FOXM1 played important roles in paclitaxel sensitivity,^34^ and modulated the segregation of chromosome in mitosis by regulating the expression of KIF20A,^11^ CENP-A, B, F^35, 36^ and its potential downstream target bicaudal D2 (BICD2).^37^ At mRNA levels, we found that DT-13/NVB co-treatment significantly reduced the expression of FOXM1 and BICD2, compared with DT-13 or NVB treatment alone (Figures 5a and b). Meanwhile, other downstream targets of FOXM1 showed no great changes in both cells (Supplementary Figures 4A–D). Subsequently, western blotting analysis showed that FOXM1 expression at 48 h, BICD2 expression at both 12 and 48 h was dramatically decreased by combination treatment (Figure 5c). However, we found that FOXM1 expression at 12 h was not significantly changed (Figure 5c). Previous studies showed that FOXM1 could be increased at the post-translational levels by the treatment of mitotic inhibitors.^34, 35, 36, 37, 38, 39^ Hence, we performed nuclear and cytoplasmic protein extraction analysis and found that DT-13/NVB co-treatment strongly inhibited the FOXM1 expression in the nucleus (Figure 5d). DT-13 also exhibited inhibitory effects on FOXM1 expression in cytoplasm and nucleus in a time-dependent manner, whereas NVB triggered upregulation of FOXM1 expression before downregulation (Supplementary Figure 5A). In addition, DT-13 significantly decreased FOXM1 and BICD2 expression in a dose-dependent manner (Supplementary Figure 5B).

### FOXM1 regulated the expression of BICD2, whereas deletion furtherly potentiated the mitotic spindle defects induced by NVB

**Figure 6:**
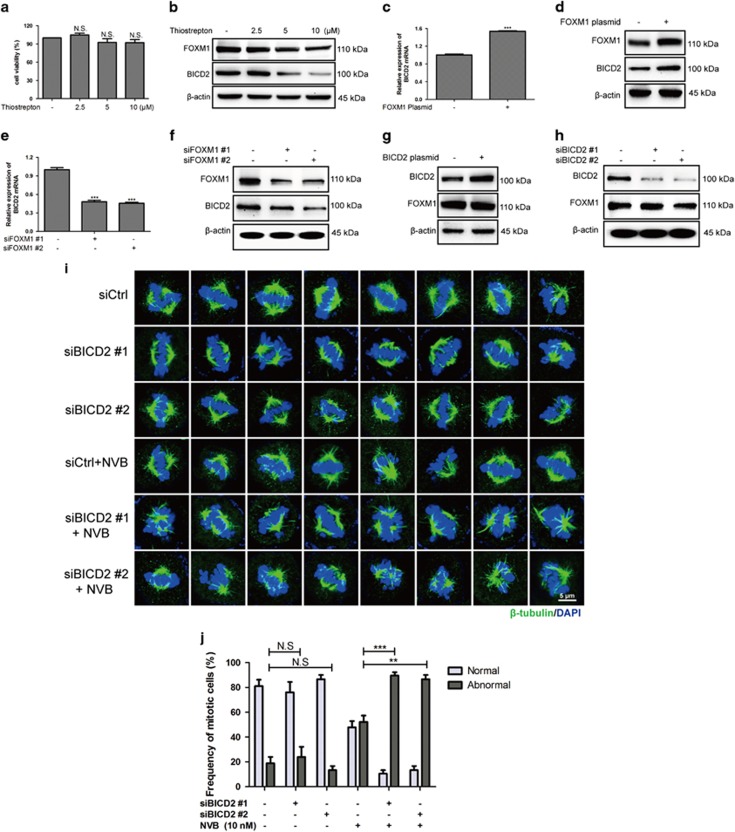
FOXM1 positively regulated BICD2 expression, and BICD2 downregulation enhanced NVB-induced defects of the mitotic spindle. (**a**) The viability of NCI-H460 cells after thiostrepton treatment for 24 h was determined by MTT assay. (**b**) Effects of thiostrepton on the expression of FOXM1 and BICD2 for 24 h were analyzed by western blotting, and *β*-actin was served as the loading control. (**c–f**) BICD2 expression at mRNA and protein level was detected by altering the levels of FOXM1 in NCI-H460 cells by transfecting FOXM1 plasmid or siRNAs. (**g, h**) FOXM1 protein levels were analyzed by transfecting BICD2 plasmid or BICD2 siRNAs. (**i**) Mitotic spindle defects were observed by immunofluorescence assay in NCI-H460 cells following BICD2 deletion with or without 0.01 *μ*M NVB treatment. Cells were immunostained with the antibody against *β*-tubulin (green) and the nucleus was stained with DAPI (blue), the mitotic cells were observed by confocal microscopy (× 100 magnification). (**j**) For each sample, at least 30 mitotic cells were captured in images. The data were expressed as mean±S.D. in triplicate using Student’s *t*-test (two-tailed). **P<0.01 and ***P<0.001. NS, no significant changes

Effects of DT-13 or NVB treatment alone revealed that FOXM1 and BICD2 expression displayed similar kinetic changes in both NCI-H460 and A549 cells (Supplementary Figures 5A–C). To test the relationship between FOXM1 and BICD2, we used the FOXM1 inhibitor thiostrepton, which inhibited the transcription of FOXM1.^40^ Here, we found that thiostrepton decreased FOXM1 levels at non-cytotoxicity concentrations (Figures 6a and b). Consistently, BICD2 expression was also decreased by thiostrepton treatment in a dose-dependent manner (Figure 6b). Furthermore, overexpression of FOXM1 significantly increased BICD2 expression at mRNA and protein levels (Figures 6c and d). Likewise, FOXM1 deletion by siRNAs also decreased BICD2 expression (Figures 6e and f). To further determine whether FOXM1 is an upstream activator of BICD2, we found that overexpression or deletion of BICD2 did not change the protein levels of FOXM1 (Figures 6g and h).

As reported, FOXM1 deletion by siRNAs triggered mitotic spindle defects.^11^ However, there was no data about the effects of BICD2 in mitotic spindle formation or chromosome alignment. In our study, we found that BICD2 deletion by siRNAs showed no significant changes in mitotic spindle formation, but potentiated NVB-induced mitotic spindle defects in NCI-H460 cells (Figures 6i and j). Collectively, these data indicated that FOXM1 is an upstream activator of BICD2 expression, and BICD2 deletion was an amplification signal of NVB-induced mitotic defects.

### Levels of FOXM1 or BICD2 were related to mitotic arrest induced by DT-13/NVB co-treatment

**Figure 7:**
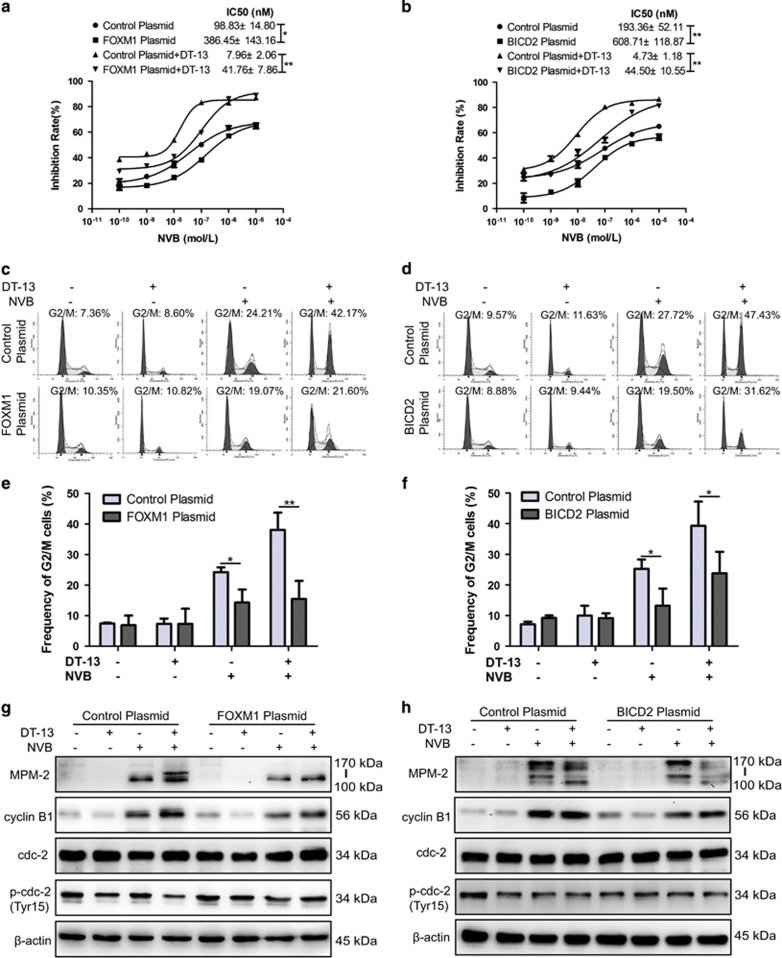
Synergistic effect induced by combination treatment was reversed by the overexpression of FOXM1 or BICD2 in NCI-H460 cells. (**a**, **b**) NCI-H460 cells were treated with 10 *μ*M DT-13 and indicated concentration of NVB for 48 h after transfecting FOXM1 or BICD2 plasmid, and cell viability was determined by MTT assay. (**c**, **d**) NCI-H460 cells were treated with 10 *μ*M DT-13 and 0.01 *μ*M NVB for 12 h after transfecting FOXM1 or BICD2 plasmid, and the extent of mitotic arrest induced by combination treatment was determined by flow cytometry. (**e**, **f**) Percentages of cells arrested in G2/M phase were shown in the histograms. (**g, h**) Expression of MPM2, cyclin B1, cdc2 and phosphorylation of cdc2 (Tyr15) was determined by western blotting analysis, *β*-actin was served as the loading control. The data were expressed as mean±S.D. in triplicate using Student’s *t*-test (two-tailed). *P<0.05 and **P<0.01

In order to further determine whether FOXM1-BICD2 regulation axis was involved in DT-13/NVB-induced synergistic effects, cells were transfected with FOXM1 and BICD2 plasmids, respectively. As shown in Figure 7a and Supplementary Figure 6A, we found that co-treatment-induced cytotoxicity was greatly reduced after FOXM1 overexpression, and NVB sensitivity was also decreased in both NCI-H460 and A549 cells. Importantly, BICD2 overexpression also exhibited similar results in drug sensitivity induced by NVB alone or combination treatment in both NCI-H460 and A549 cells (Figure 7b; Supplementary Figure 6B). Meanwhile, the results of propidium iodide (PI) staining revealed that DT-13/NVB-induced mitotic arrest was significantly reversed after FOXM1 overexpression, compared with strong synergistic effect in negative controls (Figures 7c and e; Supplementary Figures 6C and 6E). Likewise, overexpression of BICD2 also reduced the percentages of cells arrested in mitosis (Figures 7d and f; Supplementary Figures 6D and 6F). At protein levels, we found that MPM2 expression and cyclin B1/cdc2 activation were significantly decreased in combination group by overexpression of FOXM1 or BICD2 (Figures 7g and h; Supplementary Figures 6G and 6H). Furthermore, we found that drug sensitivity induced by NVB alone or combination treatment was all increased by FOXM1 or BICD2 siRNAs (Supplementary Figures 7A and 7B). Unexpectedly, we found that mitotic arrest was attenuated, but apoptosis was simultaneously induced by combination treatment for 12 h after FOXM1/BICD2 deletion according to strong PARP cleavage (Supplementary Figures 7C and 7D). Previous study had revealed that the prolonged mitotic arrest could trigger apoptosis. Here, we found that mitotic arrest induced by combination treatment was greatly enhanced at shorter-term treatment for 8 h (Supplementary Figures 7E and 7F), which further demonstrated the important roles of both genes in mitosis progression. Taken together, these results demonstrated our hypothesis about the involvement of FOXM1 and BICD2 in DT-13-/NVB-induced synergistic effects.

### Combination treatment exhibited synergistic antitumor effects *in vivo*

**Figure 8:**
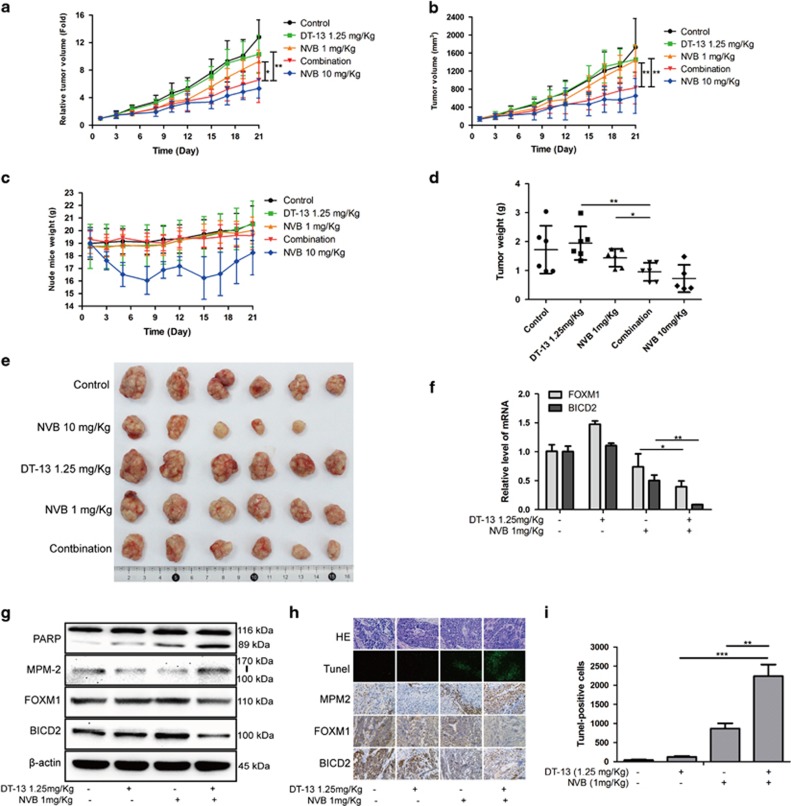
DT-13 and NVB cooperated to suppress tumor growth in NCI-H460 xenograft nude mice. Statistical analysis of relative tumor volume (**a**), tumor volume (**b**), nude mice weight (**c**) and tumor weight (**d**) was performed, and 10 mg/kg NVB was used as a positive control. (**e**) Image of resected xenograft tumor after combination treatment was shown. (**f**) Relative mRNA levels of FOXM1 and BICD2 from tumor tissue were determined by RT-qPCR analysis. (**g**) The protein levels of PARP, MPM2, FOXM1 and BICD2 were examined by western blotting, and *β*-actin was served as the loading control. (**h**) Tumor tissues from NCI-H460-xenografted nude mice after DT-13/NVB treatment were performed by TUNEL assay. Molecular alterations of MPM2, FOXM1 and BICD2 in each group were detected by immunohistochemistry analysis. (**i**) The quantity of tunnel-positive cells in each visual field was statistically analyzed in the histograms. The data were expressed as mean±S.D. using Student’s *t*-test (two-tailed). *P<0.05, **P<0.01 and ***P<0.001. RT-qPCR, real-time quantitative PCR

The above data revealed that DT-13 synergistically enhanced NVB sensitivity *in vitro*. Here, we performed an animal experiment to evaluate the combinational effects in NCI-H460-xenografted model. After 21 days treatment, we found that 1.25 mg/kg DT-13 and 1 mg/kg NVB showed no effective inhibition on the growth of NCI-H460 xenograft. However, combination treatment exhibited strong inhibitory effects on relative tumor volume (RTV) and tumor volume (TV) (Figures 8a and b). Furthermore, DT-13/NVB co-treatment did not trigger obvious toxicity *in vivo* according to the similar nude mice weight (Figure 8c). Analysis of resected xenograft tumor size and weight further determined the synergistic effects of DT-13 and NVB *in vivo* (Figures 8d and e). To confirm the molecular mechanisms of the combination treatment *in vitro*, we performed related detection in tumor tissues. Interestingly, both of FOXM1 and BICD2 were significantly decreased at mRNA and protein levels after DT-13/NVB co-treatment (Figures 8f and g). Western blotting analysis showed that apoptosis and mitotic arrest were simultaneously induced by the combination treatment according to PARP cleavage and MPM2 expression (Figure 8g). In addition, in the tumor tissues of combination group, the expression of MPM2 was also increased, whereas FOXM1 and BICD2 were reduced (Figure 8h), and the quantity of Tunel-positive cells was dramatically induced by combination treatment (Figures 8h and i). These data showed that DT-13/NVB treatment exhibited synergistic inhibitory effects on the growth of NCI-H460 xenograft, and boosted the mitotic arrest and apoptosis via inhibition of FOXM1 and BICD2, which was in accordance with the mechanisms *in vitro*.

Furthermore, the combinational effects of DT-13 and NVB were also evaluated in A549 xenograft nude mice. Compared with DT-13 or NVB treatment alone, DT-13/NVB co-treatment exhibited significant inhibitory effects on the RTV of A549 xenograft, while there was no obvious toxicity *in vivo* according to the similar changes of nude mice weight (Supplementary Figures 8A and 8B). Analysis of the resected xenograft tumor size furtherly determined the synergistic effect of DT-13 and NVB *in vivo* (Supplementary Figures 8C and 8D). In our initial work, we found that DT-13 also synergistically enhanced the cytotoxicity of the anti-mitosis drug taxol in NSCLC cells. Here, our study also demonstrated that 1.25 mg/kg DT-13 combined with 5 mg/kg taxol exhibited synergistic inhibitory effects on the RTV and tumor weight in NCI-H1975 xenograft nude mice, while the nude mice weight showed no significant changes (Supplementary Figures 8E–H).

Taken together, DT-13 significantly enhanced the antitumor effects of NVB or taxol *in vivo*, which provided competent evidence to confirm that DT-13 might provide an effective strategy for the chemosensitization of NVB or taxol in the clinical therapy of NSCLC.

## Discussion

In our present study, we determined a feasible synergistic combination treatment of DT-13 and MTAs to inhibit the proliferation of NSCLC cells *in vitro* and *in vivo*. NVB was screened out from multiple first-line chemotherapeutic drugs in NSCLC and showed higher effectiveness when combined with DT-13 together in NSCLC cells that express wild-type EGFR. Foremost, the synergistic effects were confirmed by calculation of CI values and inhibition of colony formation. Subsequently, mitotic arrest and apoptosis were triggered by DT-13/NVB co-treatment at different time, and mitotic arrest was further confirmed to be required for apoptosis induction. Importantly, transcription factor FOXM1 was identified as the upstream regulator of motor adaptor BICD2, and this axis was further demonstrated to be correlated with the synergistic effectiveness.

Cell cycle progression of eukaryote cells is tightly controlled by several checkpoint complex, among which mitosis-promoting factor (MPF) consisted of cyclin B1 and cdc2 kinase plays important roles in G2/M transition. Cyclin B1 accumulation and cdc2 activation through dephosphorylating the residues Thr 14 and Thr 15 were necessary for the initiation of mitosis progression.^41^ Here, our results showed that combination treatment triggered MPF activation through upregulating cyclin B1 levels and decreasing the phosphorylation of cdc2 at short-term treatment (Figure 3c). Moreover, cyclin B1 was reported to be destructed by anaphase-promoting complex/cyclosome just after SAC was inactivated, which was needed for anaphase initiation.^7, 42^ As expected, we found that cyclin B1 expression was greatly decreased by long-term treatment, and mitotic arrest was required for apoptosis induction (Figure 4). On the basis of the effectiveness of NVB triggering SAC activation,^28^ we concluded that NSCLC cells treated by combination treatment were arrested in prophase because of the sustained SAC activation.

To unravel the molecular mechanisms, we focused our attention on transcription factor FOXM1, which showed potent transcription regulator activity on various downstream targets in cell cycle progression. FOXM1 was expressed in almost all embryonic tissues, especially in highly proliferative cells from epithelial and mesenchymal tissues.^43, 44^ Moreover, FOXM1 expression was involved in the drug sensitivity and resistance of paclitaxel.^11, 13^ However, there is no data regarding effects of FOXM1 expression on the sensitivity of microtubule-destabilizing agents before, and our present study showed that FOXM1 deletion by siRNAs greatly enhanced NVB-induced inhibition of cell proliferation (Supplementary Figure 7A). In contrast, FOXM1 overexpression significantly increased the IC50 of NVB and attenuated the G2/M arrest induced by NVB alone (Figures 7a and e). Meanwhile, we found that DT-13/NVB co-treatment greatly reduced nuclear FOXM1 levels, and we further demonstrated that downregulation of FOXM1 expression was related to the synergistic effectiveness.

In prophase progression, SAC was served as a mechanism to prevent chromosome missegregation and aneuploidy formation by regulating correct attachment of microtubule to kinetochores.^7^ Traditional mitosis-targeting drugs, such as taxanes and vinca alkaloids, directly interfered with the polymerization or depolymerization of microtubules to activate SAC and induce mitotic arrest.^14^ Moreover, microtubule-associated proteins (MAPs) also showed activities to alter microtubule dynamics and played great roles in tumorigenesis and tumor development.^45^ Collectively, MAPs included oncogenes, tumor suppressors, apoptosis regulators and microtubule motor proteins, and showed potency to influence the effectiveness of MTAs.^45, 46^ Generally speaking, microtubule-stabilizing proteins, such as MAP1, 2, 4, 7, Tau and VHL, promoted the effects of microtubule-stabilizing taxanes, whereas microtubule-destabilizing proteins, such as stathmin, XKCM1 and TFC-D, increased the effects of microtubule-destabilizing vinca alkaloids.^45^

In mitosis progression, KIF20A, a downstream target of FOXM1, was a motor protein from kinesin-6 subfamily, which regulated separation of spindle poles and was related to paclitaxel-induced mitotic catastrophe and cellular senescence.^11^ Furthermore, CENP-A, CENP-B and CENP-F^35, 36^ were also reported to be regulated by FOXM1 and modulate chromosomal segregation in the prophase of mitosis. In our study, we found that DT-13/NVB co-treatment showed no significant and consistent effects on these downstream targets of FOXM1 in both NCI-H460 and A549 cells (Supplementary Figure 4).

Microtubules were one of the major cytoskeleton in eukaryote cells and provided a rail for microtubule motor proteins to transport their cargos.^47^ Dyneins were microtubule minus-end-directed molecular motors involved in multiple crucial fundamental processes including vesicles transportation and mitosis.^48^ Importantly, dyneins played crucial roles in removing mitotic checkpoint components before SAC was inactivated by the correct attachment of plus-end microtubules to kinetochores.^7^ As reported, most dynein activities required the combination with dynactin, and this interaction was strongly maintained by BICD2, which is an evolutionarily highly conserved motor adaptor and involved in dynein-dependent cargos trafficking in *drosophila* and mammals.^49^ In our study, we found that the combination of DT-13 and NVB significantly reduced BICD2 expression at mRNA and protein levels. Interestingly, BICD2 exhibited the similar kinetics with FOXM1 in both NCI-H460 and A549 cells under different drug treatment, such as thiostrepton, DT-13 or NVB. To confirm our speculation that FOXM1 might be a crucial upstream regulator of BICD2 expression, we demonstrated that BICD2 expression was determined by FOXM1 levels, whereas FOXM1 expression was not influenced by the changes of BICD2 expression (Figures 6c–h). Moreover, we found that BICD2 deletion alone showed no significant effects on mitotic spindle formation, but strongly potentiated the mitotic spindle defects that were induced by NVB treatment (Figures 6i and j). In addition, overexpression of BICD2 significantly attenuated the effectiveness of NVB alone or combination treatment in both NCI-H460 and A549 cells (Figure 7b; Supplementary Figure 6B). Therefore, we concluded that the reduction of BICD2 induced by DT-13/NVB co-treatment dramatically influenced the activities of dynein and the subsequent SAC inactivation. Hence, SAC was strongly prolonged by DT-13 on the basis of SAC activation triggered by NVB treatment. These results further demonstrated the important roles of BICD2 in maintaining the interaction of dynein and dynactin, which could remove the mitotic checkpoint components to inactivate SAC signaling.

In summary, our study confirmed that DT-13 synergistically enhanced NVB-induced mitotic arrest and subsequent apoptosis in NSCLC cells. Most importantly, FOXM1-BICD2 signaling axis was confirmed to be involved in NVB sensitivity and the synergistic effects of DT-13/NVB co-treatment. Moreover, *in vivo* study revealed that DT-13 combined with NVB significantly suppressed tumor growth in nude mice xenograft model, meanwhile, the changes of FOXM1 and BICD2 in tumor tissues further demonstrated the molecular mechanisms *in vitro*. Our research indicated that targeting FOXM1 and motor adaptor BICD2 is an effective strategy to sensitize NVB, and DT-13 might be a practical candidate agent for the adjuvant chemotherapy of microtubule-interfering agents in NSCLC.

## Materials and methods

### Chemicals and reagents

DT-13 was derived from Liriope muscari, and supplied by Tianjin Tasly Pharmaceutical Co., Ltd (Tianjin, China). NVB was obtained from J&K chemical (Shanghai, China). 3-(4, 5-dimethylthiazol-2-yl)-2, 5-diphenyltetrazolium bromide (MTT) and crystal violet were purchased from Sunshine Biotechnology Ltd (Nanjing, China). Apoptosis detection kit (Annexin V-PI Staining) and TUNEL detection kit were purchased from Vazyme Biotech Co., Ltd (Nanjing, China). Cell cycle detection kit (PI staining) and Nuclear Extract Kit were obtained from Beyotime Biotechnology (Shanghai, China). zVAD.fmk was purchased from MCE (MedChem Express, Princeton, NJ, USA).

### Cell culture

Human lung cancer NCI-H460, A549, NCI-H1975 and HCC827 cells were obtained from Cell Bank of Shanghai Institute for Biological Sciences, University of Chinese Academy of Sciences. Both cells were authenticated by short tandem repeat analysis to exclude possible contamination. Cells were cultured in RPMI-1640 medium (Gibco, Grand Island, NY, USA), 10% fetal bovine serum (FBS, PAN Biotech, Aidenbach, Germany) and supplemented with 100 U/ml penicillin and 100 pg/ml streptomycin. Cells were incubated in a humidified atmosphere (BB15 incubator, Thermo, Langenselbold, Germany) with 5% CO_2_ at 37 °C.

### Cell viability assay

Effects of DT-13/NVB co-treatment on NSCLC cells were determined by MTT assay. Cell suspensions were prepared and 2000 cells were seeded into 96-well plate. After incubation for 24 h, cells were treated with DT-13 or NVB alone or in combination for another 48 h. Subsequently, 20 *μ*l MTT (0.5 mg/ml) was added into each well and incubated for another 4 h, and the cell supernatant was discarded and replaced with 150 *μ*l DMSO to dissolve the formazan precipitate. The absorbance was detected at 570 nm using a Universal Microplate Reader (Infinite M100, Tecan, Germany) The inhibition rate was calculated by the formula: Inhibition rate (%)=(1-Absorbance of the treated group/Absorbance of the control group) × 100.

### Colony formation assay

Effect of combination treatment on cell proliferation was detected by colony formation assay. About 500 cells were seeded into six-well plate and incubated for 24 h. Subsequently, cells were treated with 10 *μ*M DT-13 and 0.01 *μ*M NVB alone or in combination for 12 h. Cells were then cultured in drug-free medium for another 8 days before fixation with 0.5% crystal violet and staining with 4% formaldehyde. The number of colonies was then counted macroscopically.

### Apoptosis detection

Induction of apoptosis was determined by Annexin V/PI staining. Cells were collected with EDTA-free trypsin, and washed with ice-cold PBS for two times. Subsequently, cells were suspended with 500 *μ*l binding buffer, and stained with 5 *μ*l PI and 5 *μ*l FITC-conjugated Annexin V for 15 min. Apoptotic cells were analyzed by FACSCalibur flow cytometry (BD Biosciences, San Jose, CA, USA).

### Western blotting analysis

Western blotting analysis was performed as the previous study.^19^ Antibodies used were as following: rabbit polyclonal anti-PARP, rabbit polyclonal anti-caspase-3, rabbit polyclonal anti-cleaved caspase-3, rabbit polyclonal anti-caspase-9, rabbit polyclonal anti-cleaved caspase-9, rabbit polyclonal anti-cyclin B1, mouse polyclonal anti-cdc2 and rabbit polyclonal anti-p-cdc2 (Tyr15) were purchased from Cell Signaling Technology (Beverly, MA, USA). Mouse polyclonal anti-MPM2 and rabbit polyclonal anti-BICD2 were commercially available from Millipore Corporation (Bedford, MA, USA), and rabbit polyclonal anti-FOXM1 antibody was purchased from ABclonal Technology (Wuhan, China). Mouse polyclonal anti-*β*-actin was purchased from Sunshine Biotechnology Ltd. Goat polyclonal anti-rabbit IgG conjugated to HRP and goat polyclonal anti-mouse IgG conjugated to HRP (Cell Signaling Technology) were used as secondary antibodies, and enhanced chemiluminescence reagents (Millipore) was used for detection and exposed by Gel 2000 image analyzer (Bio-Rad, Richmond, CA, USA).

### Cell cycle analysis

Distribution of cell cycle was detected by PI staining. Cells were collected and fixed in 75% ethanol overnight after drug treatment. Afterwards, cells were washed with ice-cold PBS for one time and stained with PI staining for 30 min at 37 °C. Cell cycle analysis was performed using FACSCalibur flow cytometry (BD Biosciences).

### Quantitative real-time PCR

Real-time quantitative PCR is described in Supplementary Methods.

### Plasmids transfection

FOXM1 and BICD2 plasmids were purchased from Hanbio Biotechnology Co., Ltd, and had been verified by direct sequencing. Plasmids were transfected into NCI-H460 cells by using Lipofectamine 2000 (Invitrogen, Carlsbad, CA, USA), according to the manufacturer’s instructions. Cells were incubated in transfection medium for 6 h, and then incubated in complete medium for another 18 h. All experiments were then performed and repeated for three times.

### RNA interference

The transfection of siRNA against FOXM1 and BICD2 is described in Supplementary Methods.

### Immunofluorescence assay

The immunofluorescence assay was described previously.^24^ Cells were immunostained with the antibody against *β*-tubulin (Cell Signaling Technology) and the nuclei were stained with 4', 6-diamidino-2-phenylindole (DAPI, Thermo Fisher Scientific, San Jose, USA). Mitotic cells were observed by confocal laser scanning microscopy (FV10-ASW, Version 2.1, Olympus, Tokyo, Japan).

### Nude mice xenograft study

Female BALB/c athymic nude mice (5 weeks) with body weight from 18 to 22 g were purchased from the Model Animal Research Center of Nanjing University. About 3 × 10^6^ NCI-H460 cells were injected into the subcutaneous tissue of armpit. Tumor tissues were grown with a volume about 400 mm^3^, then resected and cut into small pieces. Subsequently, the pieces of tissue were planted subcutaneously into each nude mice. After 10 days, tumor sizes were measured by micrometer calipers. After excluding the mice with unsuitable tumor size, the mice with analogous TV were randomly divided into five groups with six individuals per group. DT-13 was intragastrically administrated with a concentration of 1.25 mg/kg, and NVB was intravenously administrated with dosages of 1 and 10 mg/kg (positive control). The negative group was given an equal amount of normal saline. After administration for 21 days, mice were killed, and the tumor tissues were then resected and detected. TV and RTV were calculated by the following formula: TV (mm^3^)=*A*/2 × *B*^2^, where *A* represents the longest diameter of tumor, and *B* represents the shortest diameter. RTV=*V_t_*/*V*_0_, where *V_t_* represents the TV of day *t*, and *V*_0_ represents the TV of day 0. Animal care and surgery operation were all guided by Animal Care and Control Committee in China Pharmaceutical University.

### Immunohistochemical analysis

The expression of MPM2, FOXM1 and BICD2 in tumor tissues of BALB/c nude mice was detected as previously described.^24^ Apoptotic cells in tumor tissues were determined by TUNEL BrightGreen Apoptosis Detection Kit (Vazyme Biotech Co., Ltd), according to the manufacturer’s protocol.

### Statistical analysis

Drug interactions were assessed as CI, which was calculated by CalcuSyn software program (Version 2.1, Biosoft, Cambridge, UK). CI<0.9 represents synergism, 0.9<CI<1.1 represents additivity and CI>1.1 represents antagonism. All data in the study were expressed as mean±S.D. using Student’s *t*-test (two-tailed). *P<0.05, **P<0.01, ***P<0.001 and NS represents no significant changes.
