# Supplementary material for: DT-13 synergistically enhanced vinorelbine-mediated mitotic arrest through inhibition of FOXM1-BICD2 axis in non-small-cell lung cancer cells
Source: Cell Death Dis. 2017 May 25;8(5):e2810–. doi: 10.1038/cddis.2017.218 (PMC5520732; doi:10.1038/cddis.2017.218)

**SUPPLEMENTARY METHODS**

**Quantitative real-time PCR (RT-qPCR).** Total RNA from cells and tissues were isolated by the TRIzol® Reagent, and reverse transcribed using HiScript® II Reverse Transcriptase SuperMix (Vazyme). The mRNA level was detected using SYBR® qPCR Master Mix (Vazyme). The expression of mRNA was standardized by internal control 18s mRNA, and relative mRNA level of treated group was based on control group. The primer sequences used in RT-qPCR were showed in Supplementary Table S2.

**RNA interference.** Cell transfection were performed by Lipofectamine 2000, and the operation steps were based on manufacturer’s protocol. The negative control, FOXM1 and BICD2 siRNA oligos were purchased from GenePharma (Shanghai, China). The sequences of the siRNA are as follows: FOXM1 siRNA#1, 5'- GUGUCUCGGAAAUGCUUGUTT-3' and antisense 5'-ACAAGCAUUUCCGAGACACTT-3'; FOXM1 siRNA #2, sense 5'-GGACCACUUUCCCUACUUUTT-3' and antisense 5'-AAAGUAGGGAAAGUGGUCCTT-3'. BICD2 siRNA #1, sense 5'-GCAACAAUGAGACACCCAATT-3' and antisense 5'- UUGGGUGUCUCAUUGUUGCTT-3'. BICD2 siRNA #2, sense 5'-GCCUCAAGCAUGAGAUCAATT-3' and antisense 5'-UUGAUCUCAUGCUUGAGGCTT-3'. Scrambled siRNA sense 5'- UUCUCCGAACGUGUCACGUTT -3' and antisense 5'- ACGUGACACGUUCGGAGAATT -3' was used as negative control. Cells were incubated in transfection medium for 6 h, and then incubated in complete medium for another 18h. All experiment was then performed and repeated for three time.

**Nude mice xenograft study.** Female BALB/c athymic nude mice (5 weeks) with body weight from 18 to 22 g were purchased from the Model Animal Research Center of Nanjing University. 3×106 A549 or NCI-H1975 cells were injected into the subcutaneous tissue of armpit. Tumor tissues were grown with a volume about 300 mm3, then resected and cut into small pieces. Subsequently, the pieces of tissue were planted subcutaneously into each nude mice. After 10 days, tumor sizes were measured by micrometer calipers. After excluding the mice with unsuitable tumor size, the mice with analogous tumor volume were randomly divided into five groups with six individuals per group. In A549 xenograft model, DT-13 was intragastrically administrated with a concentration of 1.25 mg/Kg, and NVB were intravenously administrated with dosages of 1 mg/Kg and 10 mg/Kg (positive control). In NCI-H1975 xenograft model, DT-13 was intragastrically administrated with a concentration of 1.25 mg/Kg, and taxol were intravenously administrated with dosages of 5 mg/Kg and 20 mg/Kg (positive control). The negative group was given an equal amount of normal saline. After the administration for more than 20 days, mice were euthanized and the tumor tissues were then resected and detected. TV and RTV were calculated by the following formula：TV (mm3) = A/2×B2, where A represented the longest diameter of tumor, and B represented shortest diameter. RTV = Vt/V0, where Vt represented the tumor volume of day t, and V0 represented the tumor volume of day 0. Animal care and surgery operation were all guided by Animal Care and Control Committee in China Pharmaceutical University.

**Statistical analysis.** Drug interactions were assessed as CI value, which was calculated by CalcuSyn software program (Version 2.1, Biosoft, Cambridge, UK). CI < 0.1 represents very strong synergism, 0.10 ≤ CI < 0.30 represents strong synergism，0.30 ≤ CI < 0.70 represents synergism，0.70 ≤ CI < 0.85 represents moderate synergism and 0.85 ≤ CI < 0.90 represents slight synergism. All data in the study were expressed as mean ± S.D. using Student’s t-test (two-tailed). *P < 0.05, **P < 0.01, ***P < 0.001, N.S. represents no significant changes.

**SUPPLEMENTARY FIGURES AND TABLES**


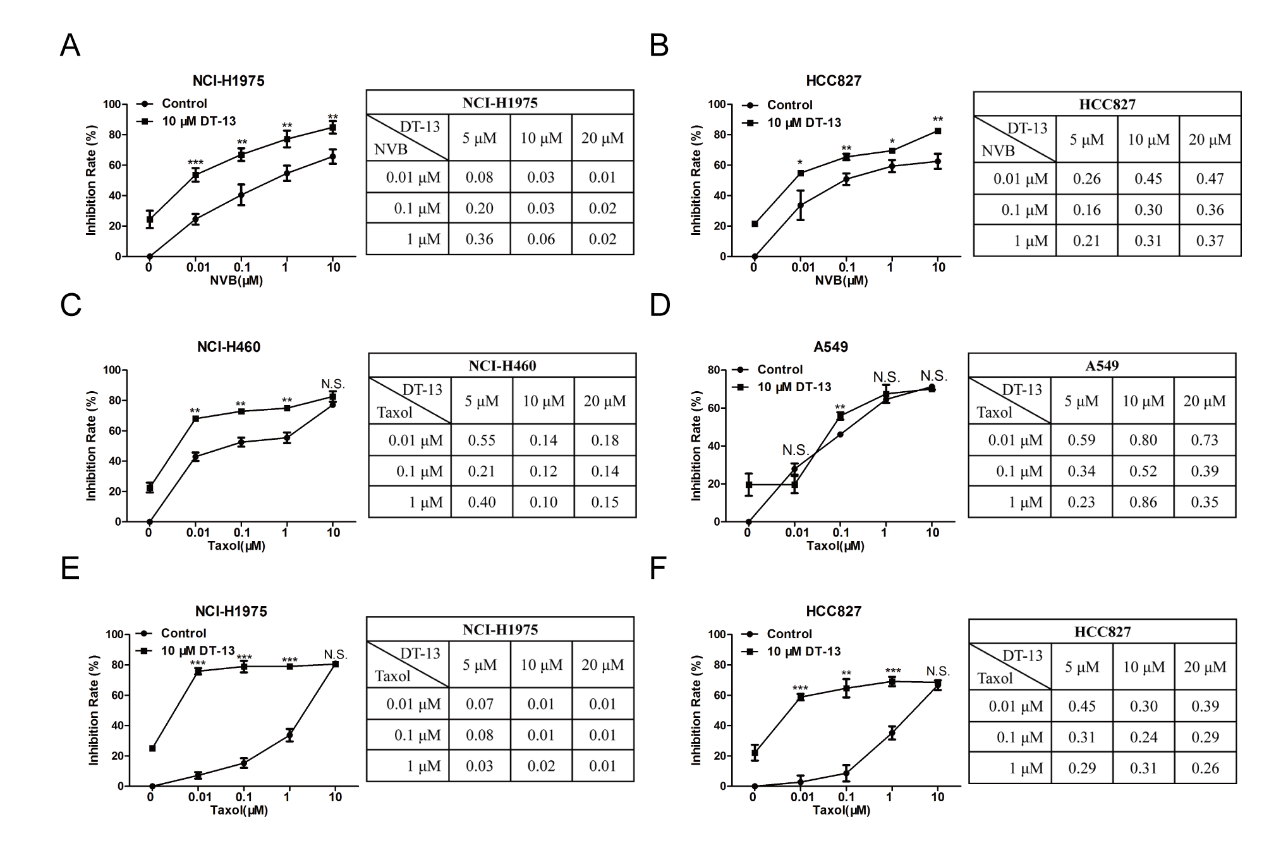


**Supplementary Figure 1.** Combinational effects of DT-13 and NVB or taxol on the viability of NSCLC cells. NCI-H1975 (A) and HCC827 (B) cells were treated with 10 μM DT-13 and indicated concentrations of NVB for 48 h. NCI-H460 (C), A549(D), NCI-H1975 (E) and HCC827 (F) cells were treated with 10 μM DT-13 and indicated concentrations of taxol for 48 h. MTT assays were performed to analyze the cell viability of NSCLC cells. CI values were calculated by CalcuSyn software, and drug interactions was indicated as synergism (CI < 0.9), additivity (0.9 < CI < 1.1) or antagonism (CI > 1.1). The data were expressed as mean ± S.D. in triplicate using Student’s t-test (two-tailed). *P < 0.05, **P < 0.01, ***P < 0.001, and N.S. represents no significant changes.


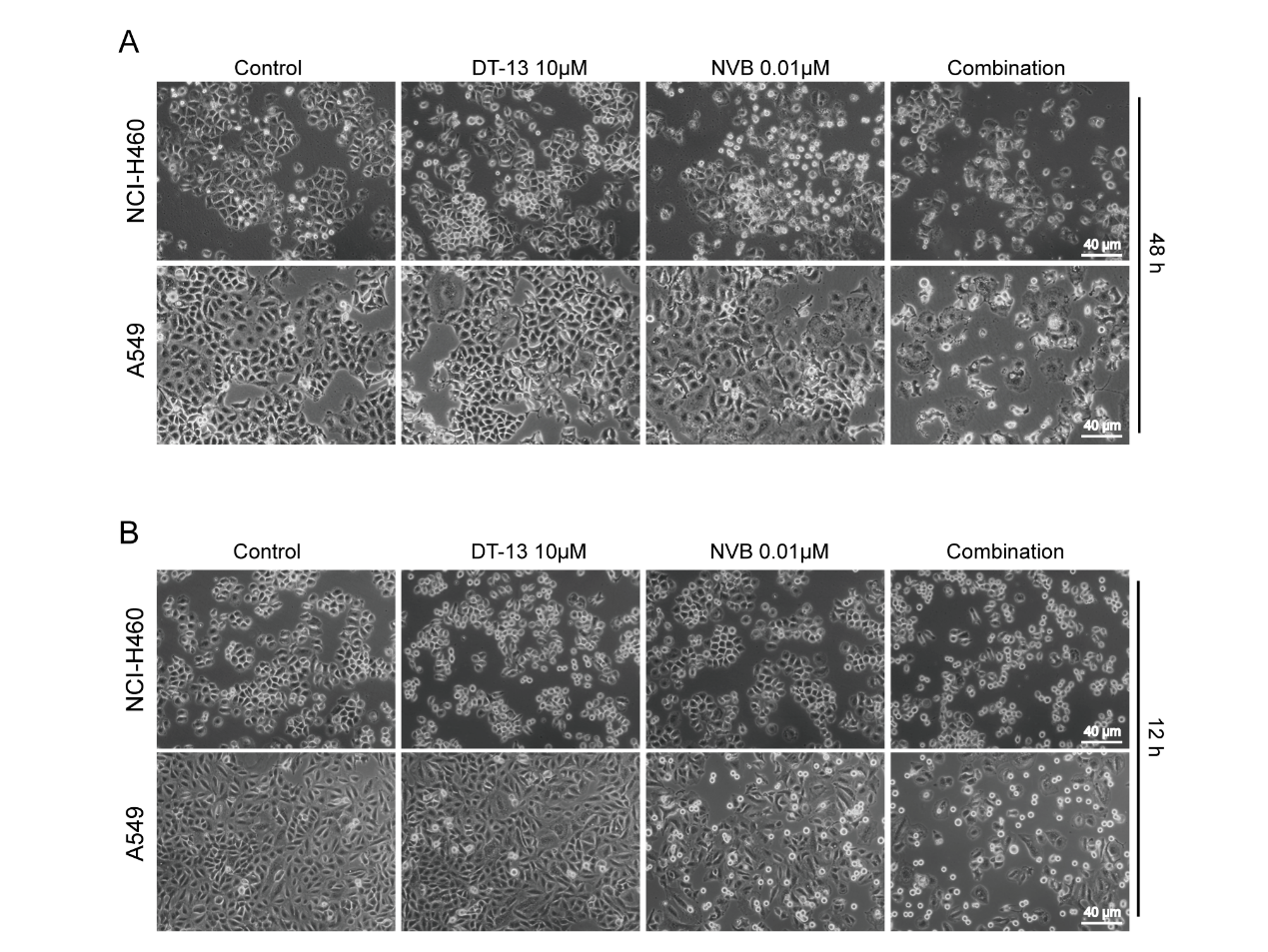


**Supplementary Figure 2.** Combination treatment triggered significant changes of cell morphology. NCI-H460 and A549 cells were treated with 10 μM DT-13 and 0.01 μM NVB， and the cell morphology at 48 h (A) and 12 h (B) was visualized by inverted microscope (Olympus IX70).


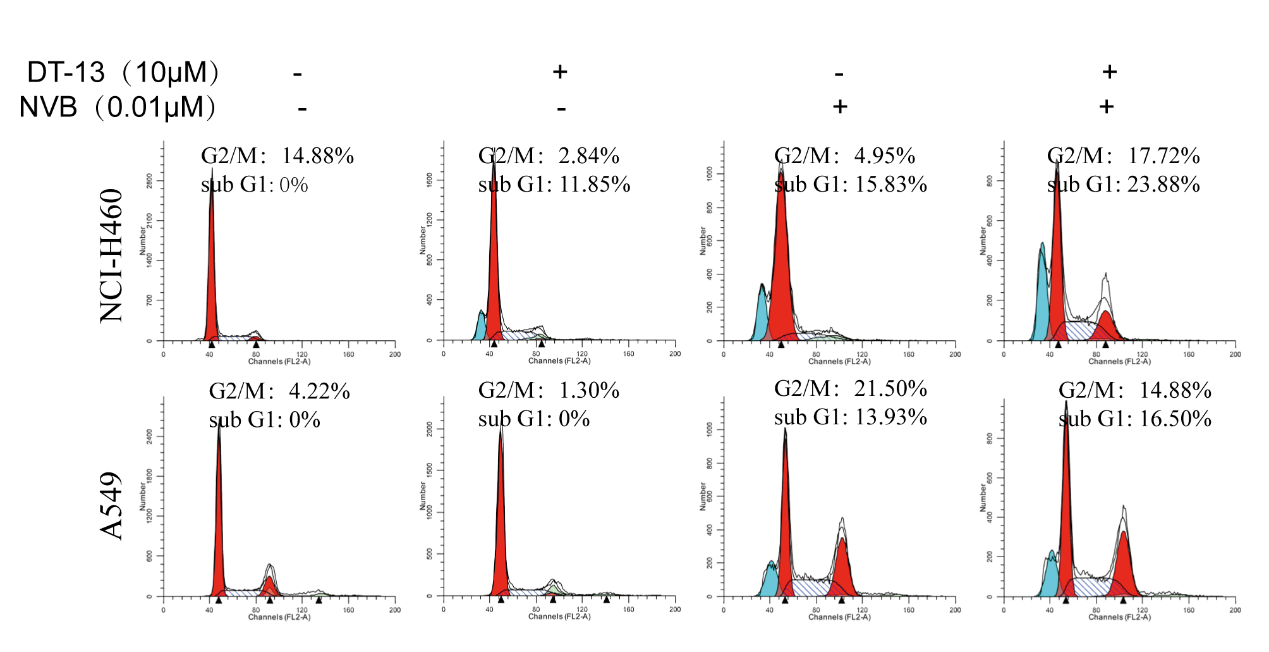


**Supplementary Figure 3.** Effects of DT-13 combined with NVB on the distribution of cell cycle at 48 h in NCI-H460 and A549 cells. NCI-H460 and A549 cells were treated with 10 μM DT-13 and 0.01 μM NVB for 48 h. Frequency of cells per phase in cell cycle was detected by flow cytometry using PI staining.


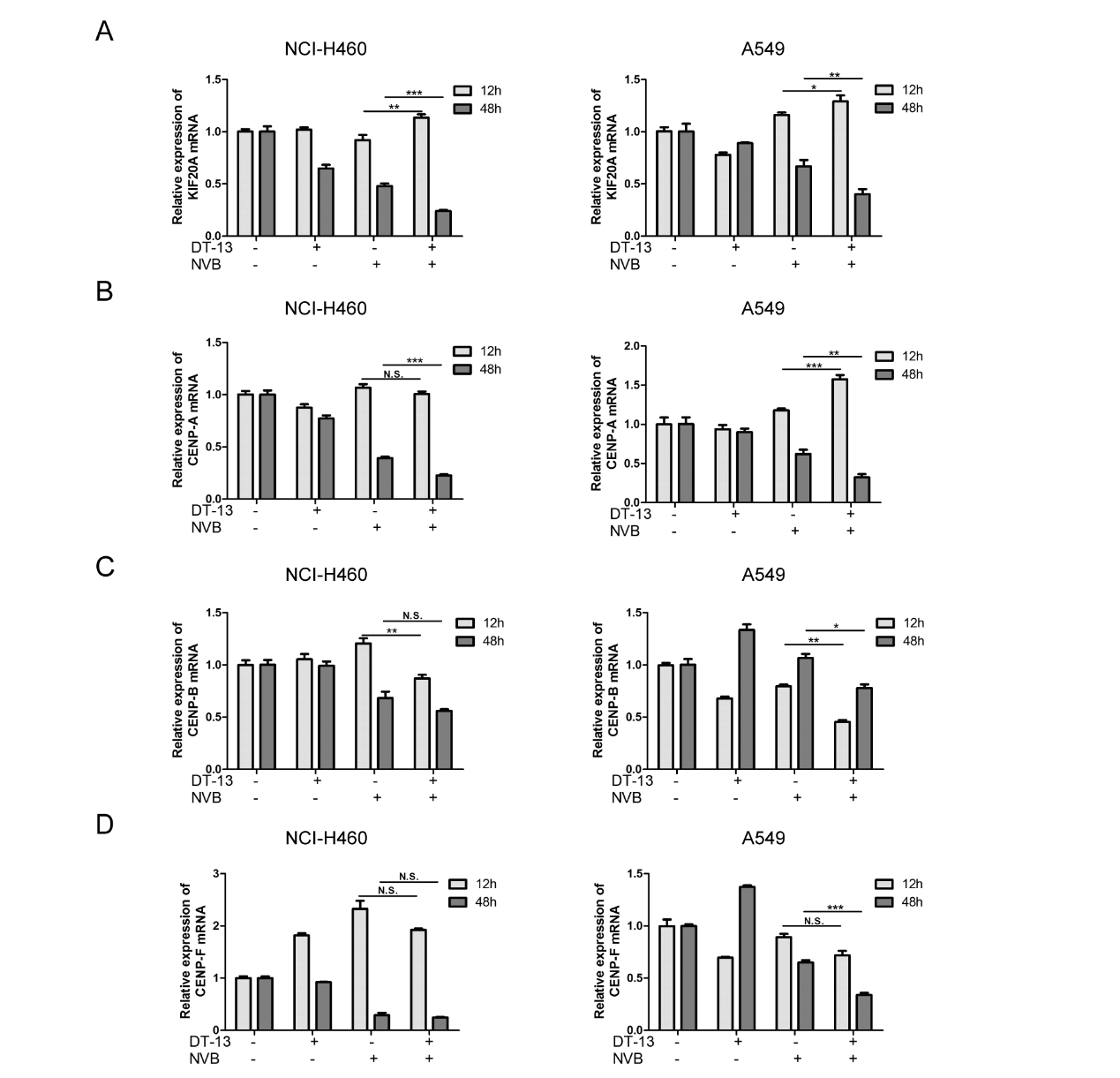


**Supplementary Figure 4.** DT-13/NVB co-treatment exhibited various effects on downstream targets of FOXM1 in mitosis. NCI-H460 and A549 cells were treated with 10 μM DT-13 and 0.01 μM NVB for 12 h and 48 h, respectively. mRNA levels of KIF20A (A), CENP-A (B), CENP-B (C) and CENP-F (D) in NCI-H460 or A549 cells were detected by RT-qPCR analysis, data were expressed as mean ± S.D. in triplicate using Student’s t-test (two-tailed). *P < 0.05, **P < 0.01 and ***P < 0.001, N.S. represents no significant changes.


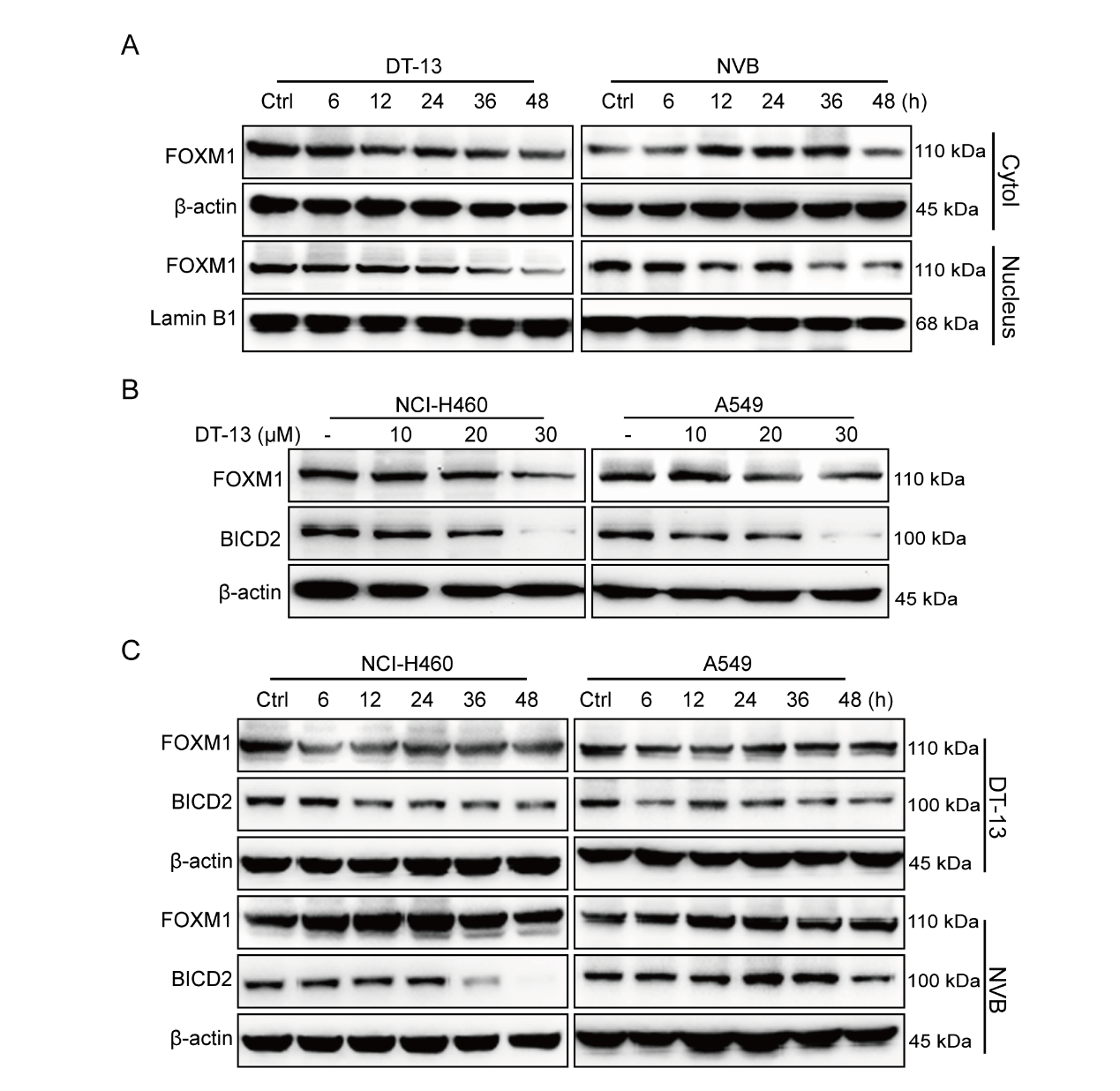


**Supplementary Figure 5.** Effects of DT-13 or NVB on the protein expression of FOXM1 and BICD2. (A). NCI-H460 cells were treated with 10 μM DT-13 or 0.01 μM NVB for indicated time. Nuclear and cytoplasmic expression of FOXM1 were detected by western blotting. Lamin B1 and β-actin were served as the loading control in nucleus and cytoplasm, respectively. (B). NCI-H460 and A549 cells were treated with indicated concentrations of DT-13 for 12 h. Total protein expression of FOXM1 and BICD2 was determined by western blotting, and β-actin was served as loading control. (C). NCI-H460 and A549 cells were treated with 10 μM DT-13 or 0.01 μM NVB for indicated time. Total protein expression of FOXM1 and BICD2 were determined by western blotting, and β-actin was served as loading control.


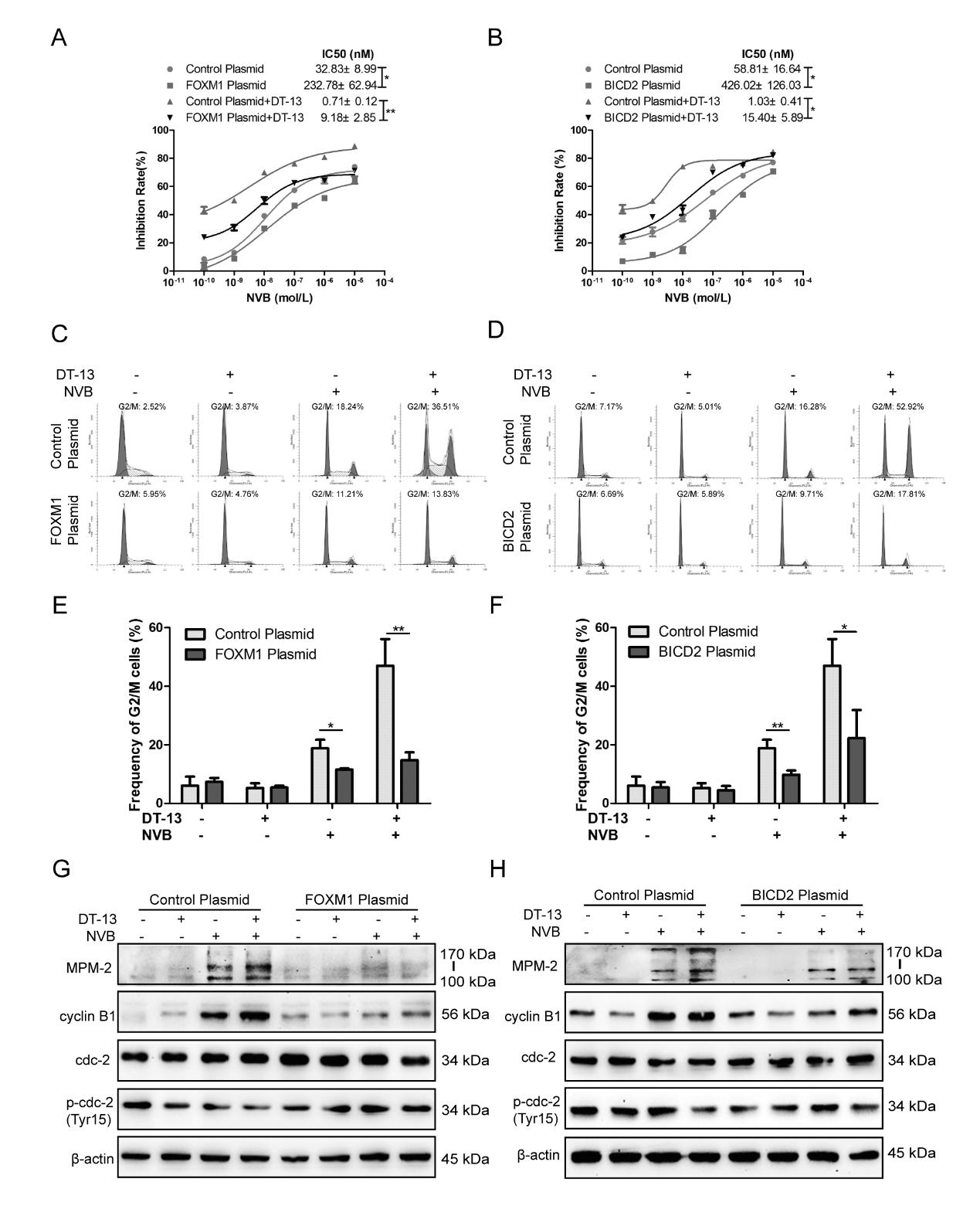


**Supplementary Figure 6.** Synergistic effect induced by combination treatment were reversed by overexpression of FOXM1 or BICD2 in A549 cells. (A, B). A549 cells were treated with 10 μM DT-13 and indicated concentration of NVB for 48 h after transfecting FOXM1 or BICD2 plasmid, and cell viability was determined by MTT assay. (C, D). A549 cells were treated with 10 μM DT-13 and 0.01 μM NVB for 12 h after transfecting FOXM1 or BICD2 plasmid, and extent of mitotic arrest induced by combination treatment was determined flow cytometry. (E, F). Percentages of cells arrested in G2/M phase were shown in the histograms. (G, H). Expression of MPM2, cyclin B1, cdc2 and phosphorylation of cdc2 (Tyr15) was determined by western blotting analysis, β-actin was served as loading control. The data were expressed as mean ± S.D. in triplicate using Student’s t-test (two-tailed). *P < 0.05 and **P < 0.01.


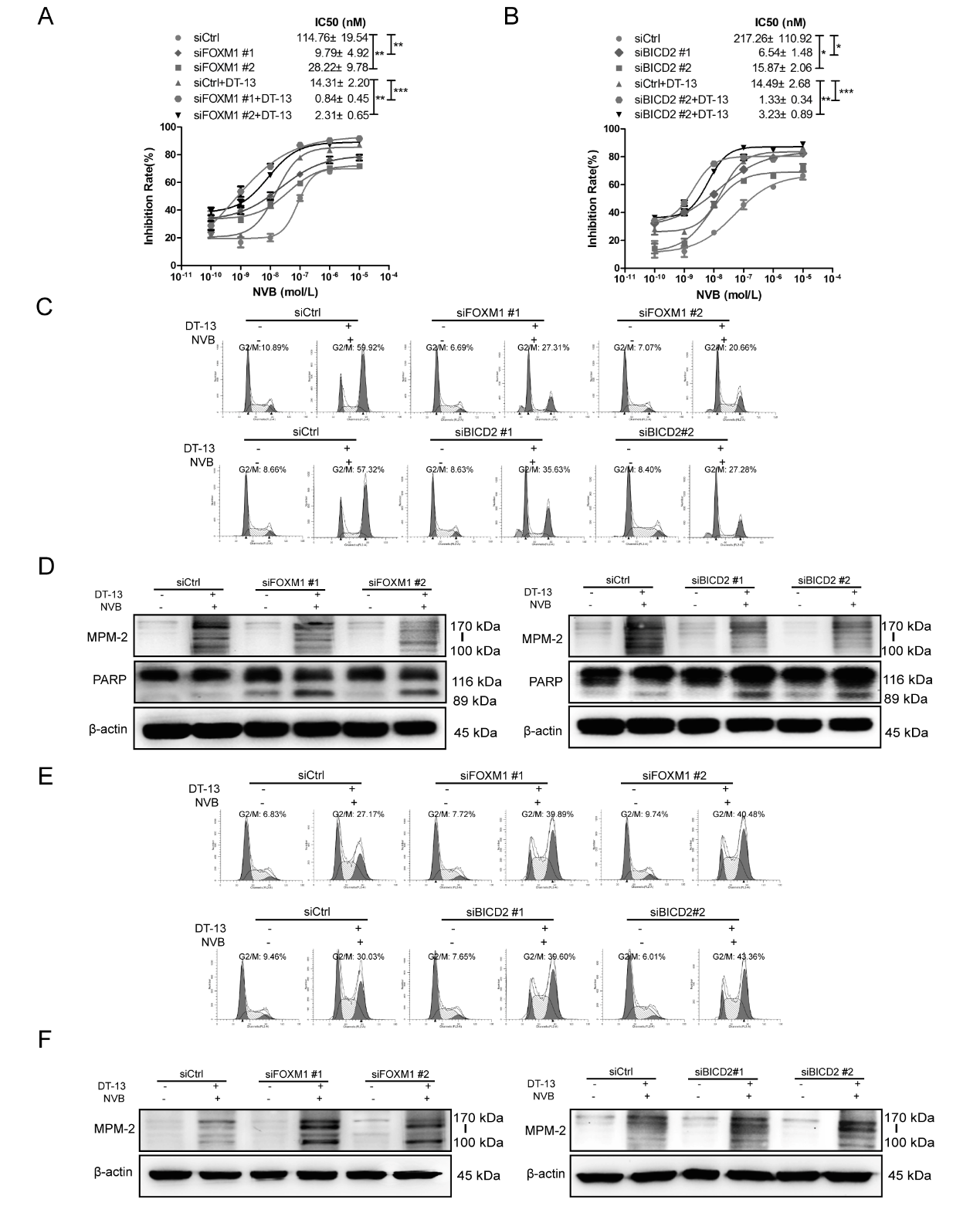


**Supplementary Figure 7.** Synergistic effect induced by combination treatment were enhanced by deletion of FOXM1 or BICD2. (A, B). NCI-H460 cells were treated with 10 μM DT-13 and indicated concentration of NVB for 48 h after transfecting FOXM1 or BICD2 siRNAs, and cell viability was determined by MTT assay. (C). NCI-H460 cells were treated with 10 μM DT-13 and 0.01 μM NVB for 12 h after transfecting FOXM1 or BICD2 siRNAs. The extent of mitotic arrest induced by combination treatment was determined by flow cytometry. (D). Expression of MPM2 and cleavage of PARP was determined by western blotting analysis, β-actin was served as loading control. (E). NCI-H460 cells were treated with 10 μM DT-13 and 0.01 μM NVB for 8 h after transfecting FOXM1 or BICD2 siRNAs, and cell cycle distribution was determined by flow cytometry. (F). Expression of MPM2 was analyzed by western blotting, β-actin was served as loading control. The data were expressed as mean ± S.D. in triplicate using Student’s t-test (two-tailed). *P < 0.05, **P < 0.01 and **P < 0.001.


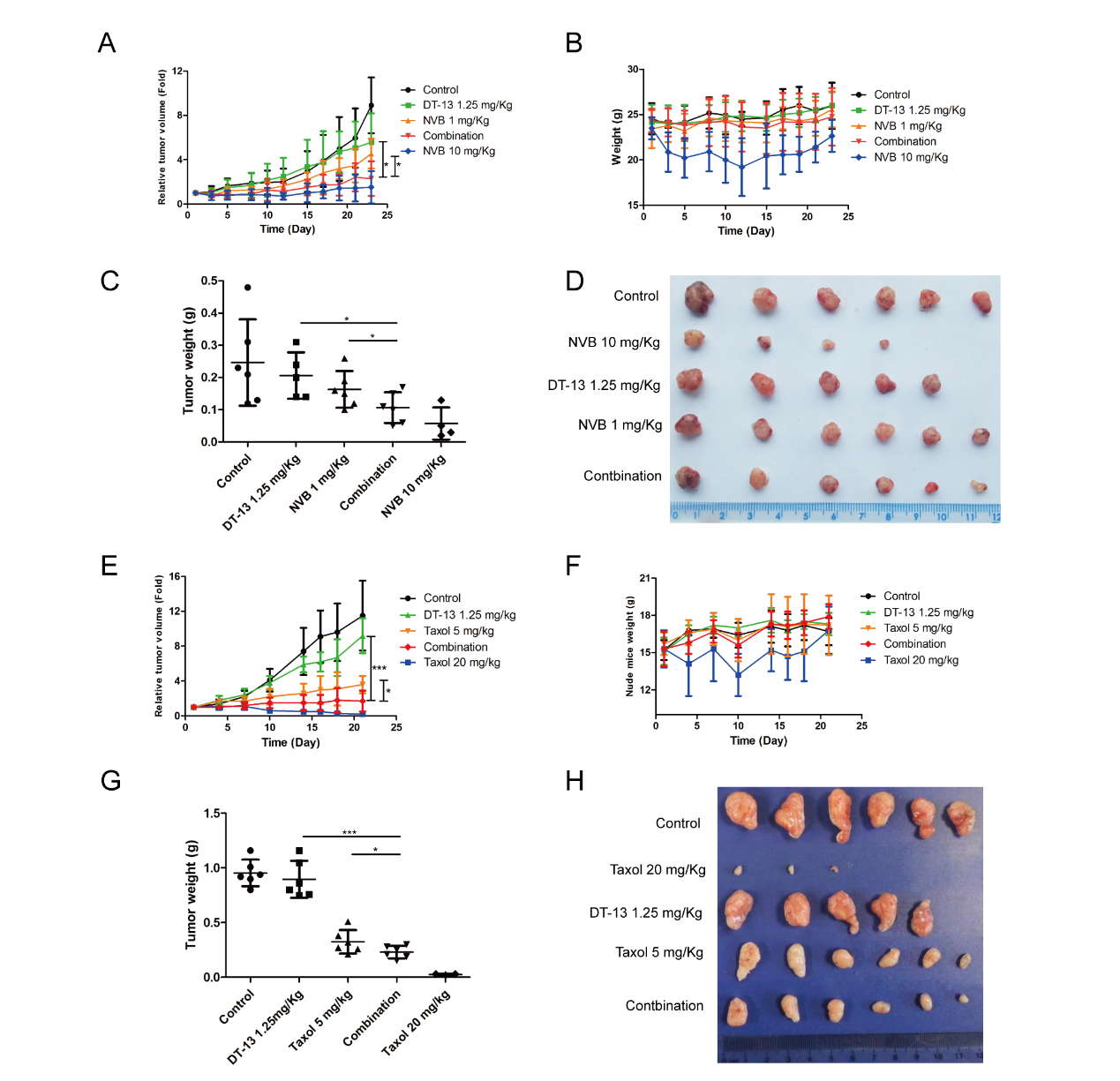


**Supplementary Figure 8.** Combination treatment of DT-13 and NVB or taxol cooperated to suppress tumor growth in A549 or NCI-H1975 xenograft nude mice. Statistical analysis of relative tumor volume (A), nude mice weight (B) and tumor weight (C) in A549 xenograft model were performed, and 10 mg/Kg NVB was used as positive control. (D). Image of resected A549 xenograft tumor after combination treatment was shown. Statistical analysis of relative tumor volume (E), nude mice weight (F) and tumor weight (G) in NCI-H1975 xenograft model were performed, and 20 mg/Kg taxol was used as positive control. (H). Image of resected NCI-H1975 xenograft tumor after combination treatment was shown. The data were expressed as mean ± S.D. using Student’s t-test (two-tailed). *P < 0.05 and ***P < 0.01.

**Supplementary Table S1:** The Summary of the synergistic effects of DT-13 combined with NVB or taxol in NSCLC cells.


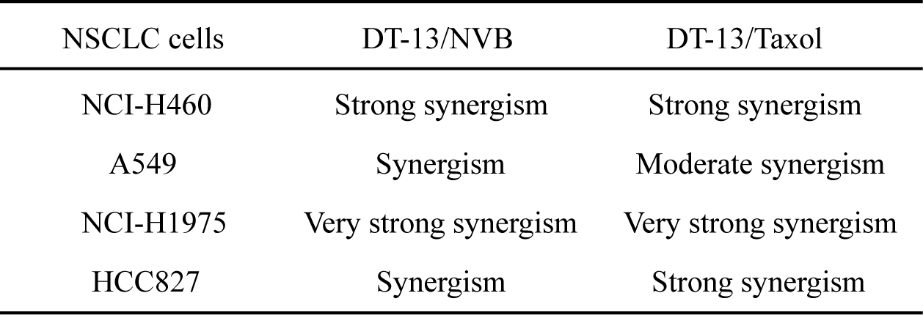


**Supplementary Table S2:** The sequences of primers used in RT-qPCR.


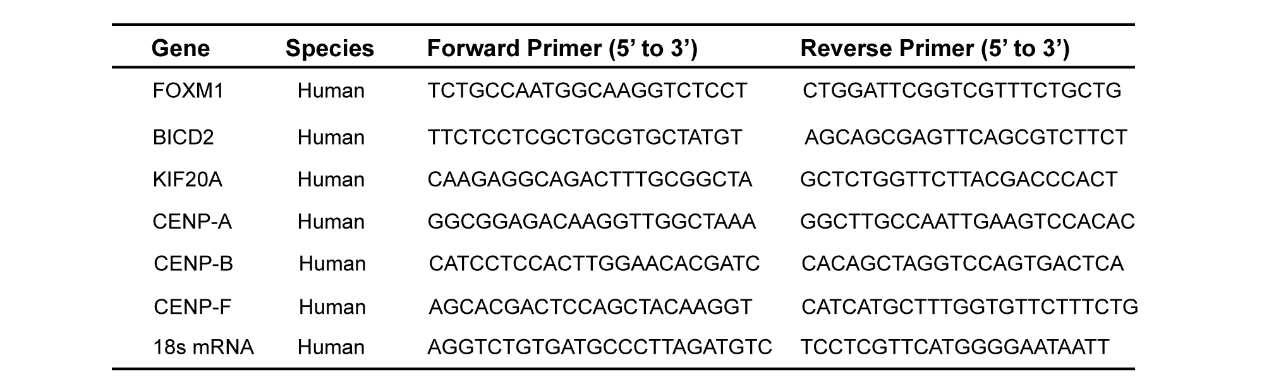

Supplement: Supplementary Information [file cddis2017218x1.docx]
